# PDGF‐induced fibroblast growth requires monounsaturated fatty acid production by stearoyl‐CoA desaturase

**DOI:** 10.1002/2211-5463.12194

**Published:** 2017-02-02

**Authors:** Alexandra Coomans de Brachène, Nicolas Dif, Audrey de Rocca Serra, Chloé Bonnineau, Amélie I. Velghe, Yvan Larondelle, Donatienne Tyteca, Jean‐Baptiste Demoulin

**Affiliations:** ^1^ de Duve InstituteMEXP unitUniversité catholique de LouvainBrusselsBelgium; ^2^ Institute of Life SciencesUniversité catholique de LouvainLouvain‐La‐NeuveBelgium; ^3^ de Duve InstituteCELL unitUniversité catholique de LouvainBrusselsBelgium

**Keywords:** oleic acid, palmitic acid, PDGFB, PDGFRB, SCD1

## Abstract

Stearoyl‐coenzyme A desaturase (SCD) catalyzes the Δ9‐cis desaturation of saturated fatty acids (SFA) to generate monounsaturated fatty acids (MUFA). This enzyme is highly up‐regulated by platelet‐derived growth factor (PDGF) in human fibroblasts. Accordingly, the analysis of cellular fatty acids by gas chromatography showed that PDGF significantly increased the proportion of MUFA, particularly palmitoleate, in cellular lipids. To further analyze the role of SCD in fibroblasts, we used small hairpin RNA targeting SCD (shSCD), which decreased the MUFA content. SCD down‐regulation blunted the proliferation of fibroblasts in response to PDGF. This was confirmed using a pharmacological inhibitor of SCD. In addition, proliferation was blocked by palmitate and stearate (two SCD substrates) but not by palmitoleate and oleate (two SCD products). In the presence of an equal amount of oleate, palmitate had no effect on cell proliferation. SCD inhibition or down‐regulation did not decrease PDGF receptor activity or signaling. However, by measuring plasma membrane lipid lateral diffusion by fluorescence recovery after photobleaching, we showed that the modulation of the MUFA/SFA ratio by PDGF and SCD inhibitor was related to modifications of membrane fluidity. Altogether, our data suggest that SCD is required for the response of normal fibroblasts to growth factors.

**Abbreviations**
MEM: minimum essential medium
MUFA: monounsaturated fatty acids
PDGF: platelet‐derived growth factor
PI3K: phosphatidylinositol 3‐kinase
SCD: stearoyl‐coenzyme A desaturase
SFA: saturated fatty acids
shSCD: small‐hairpin RNA against SCD

Growth factors, such as the platelet‐derived growth factor (PDGF), are important mediators of cellular processes such as cell proliferation, migration, survival, and differentiation 1. PDGF acts on multiple normal cell types, including fibroblasts 2, 3. PDGF ligands and their two receptors, PDGFRα and PDGFRβ, are also involved in the proliferation of cancer cells of multiple origins 1, 4, 5, 6, 7, 8. Proliferation is a complex process that requires not only DNA duplication but also increased lipid and protein synthesis. One key mediator of this cell growth program is mTOR complex 1 (mTORC1), downstream of phosphatidylinositol 3‐kinase (PI3K) and AKT (also known as PKB, Protein Kinase B) 9. mTORC1 controls protein synthesis by regulating translation and activates the sterol regulatory element‐binding protein (SREBP), a lipogenic transcription factor. This factor regulates the expression of virtually all lipogenic enzymes involved in cholesterol, fatty acid, and phospholipid synthesis, such as 3‐hydroxy‐3‐methylglutaryl‐coenzyme A synthase (*HMGCS1*), 3‐hydroxy‐3‐methylglutaryl‐coenzyme A reductase (*HMGCR*), fatty acid synthase (*FASN*), and stearoyl‐coenzyme A desaturase (*SCD*) 10, 11.

Stearoyl‐coenzyme A desaturase is an endoplasmic reticulum (ER) membrane‐associated protein that catalyzes the ∆9‐cis desaturation of saturated fatty acids (SFA) to generate monounsaturated fatty acids (MUFA) 12, 13. Its major substrates are stearoyl‐CoA (18 : 0) and palmitoyl‐CoA (16 : 0), which are converted into oleyl‐CoA (18 : 1) and palmitoleoyl‐CoA (16 : 1), respectively. These MUFA are used for the synthesis of phospholipids, triglycerides, and cholesterol esters. They are important components of membrane phospholipids and the MUFA/SFA ratio influences the plasma membrane fluidity and signal transduction 14. The expression of the human *SCD* gene is induced by growth factor stimulation via SREBP 10. This gene, which is also named *SCD1*, has up to four orthologs in rodents (*Scd1*,* Scd2*,* Scd3*, and *Scd4* in mice for instance). A second human gene, *SCD5*, is expressed only in the brain.

Several studies pointed to a role for SCD in the survival and proliferation of cancer cells 15, 16, 17, 18. We previously described a strong regulation of SCD following fibroblast treatment with PDGF, suggesting that this enzyme could also be important for normal cells 10. In the present study, we showed that SCD down‐regulation or inhibition strongly reduces normal cell proliferation in response to PDGF.

## Materials and methods

### Cells and reagents

Human foreskin fibroblasts (AG01518 and AG01523, Coriell Institute for medical research, Camden, NJ, USA) were grown in Quantum 333 complete medium (GE Healthcare, Diegem, Belgium). Experiments were performed using minimum essential medium (MEM, Thermo Fisher Scientific, Asse, Belgium). HEK293T human embryonic kidney cells (obtained from ATCC) were cultured in Dulbecco's modified Eagle's Medium (DMEM, Thermo Fisher Scientific) supplemented with 10% FBS, penicillin, and streptomycin (Thermo Fisher Scientific). The absence of mycoplasma infection was regularly checked for all cell lines using a kit from Lonza (Verviers, Belgium). PDGF‐BB was obtained from PeproTech (London, UK). SCD1 inhibitor was purchased from Gentaur (Kampenhout, Belgium, #1716‐1) and solubilized in DMSO. Sodium palmitate, sodium oleate, palmitic acid, palmitoleic acid, stearic acid, and oleic acid were obtained from Sigma (Overijse, Belgium). Fatty acid solutions were prepared as follows: fatty acids were solubilized in isopropanol, and neutralized with NaOH to obtain 20 mm solutions, which were heated at 70 °C for 30 min. Then, a solution of BSA (30% in PBS, preheated at 55 °C) was added; the mixtures were vortexed and incubated at 55 °C for 10 min. After cooling, MEM was added to obtain 0.2 mm ready‐to‐use solutions. The final BSA concentration was 5 mg/mL. Solutions containing equal amounts of isopropanol and BSA were used as control.

### Lentivirus‐mediated shRNA silencing

The design of small‐hairpin RNA (shRNA) was performed using the Whitehead Institute for Biomedical Research free software (http://sirna.wi.mit.edu). We choose the two sequences targeting human *SCD* with the highest score: AAGGCCTTTCTTCTGTGTTAA (shSCD1) and AACACATGCTGATCCTCATAA (shSCD2). We cloned them into the pLKO.1 lentiviral vector (Addgene #10878) by digestion with the restriction enzymes *Eco*RI and *Age*I. These two shRNAs were packaged for viral production and tested for target knockdown efficiency. A negative control (scramble) was obtained from Addgene (reference #1864). The production of virus and the infection of cells were performed as described previously 19, 20, 21.

### Cell lipid extraction and analysis by gas chromatography

AG01518 human fibroblasts plated in 15‐cm plates (8.10^5^ cells/plate) were either infected with lentiviral particles that express one of the small‐hairpin RNA against SCD (shSCD) or treated with PDGF‐BB (25 ng/mL) or SCD inhibitor (20 μm) for 24 h or 48 h. Cells were detached, washed in PBS, and cell pellets were stored at −80 °C. Lipid extraction and methylation were performed following Bligh and Dyer with minor modifications 22, 23. Then, fatty acid methyl esters (FAMEs) were separated, identified, and quantified by gas chromatography (Thermo Scientific Trace 1310) with a RT2560 capillary column (biscyanopropyl polysiloxane, 100 m × 0.25 mm internal diameter, 0.2‐μm film thickness, Restek, USA), a TriPlus AS auto‐sampler (Thermo Fisher Scientific) and a flame ionization detector (FID). The carrier gas was H_2_ (200 Pa, 2 mL/min). The column temperature followed this sequence: increase from 80 °C to 175 °C at 25 °C/min, hold 25 min, increase to 200 °C at 10 °C/min, hold 20 min, increase to 220 °C at 10 °C/min, hold 5 min, increase to 235 °C at 10 °C/min, hold 15 min, decrease to 80 °C at 20 °C/min. The FID was at 255 °C and received continuous flow of H_2_ (35 mL/min), N_2_ (40 mL/min), and air (350 mL/min). Identification and quantification of fatty acids was performed by comparison with retention time of pure methyl ester standards (Larodan) of known concentrations (software: ChromQuest 4.2, ThermoFinnigan).

### Proliferation assay

The measure of cell proliferation was assessed by titrated thymidine incorporation, as described previously 24, 25. Briefly, human fibroblasts were seeded in quadruplicates in a 96‐well plate in complete medium (4000 cells/well). Cells were starved in serum‐free MEM for 24 h. FBS (10%) or PDGF‐BB (25 ng/mL) was then added with [^3^H]‐thymidine (0.5 μCi/well; GE Healthcare) for 24 h. Some experiments were also performed in the presence of palmitate, palmitoleate, stearate, oleate (all at 0.2 mm), SCD inhibitor (2–10–20 μm), or vehicle. Microtiter plates were harvested using a cell harvester (PerkinElmer Life Sciences, Zaventem, Belgium). The radioactivity incorporated into DNA was quantified using a TopCount instrument (PerkinElmer Life Sciences).

### Western blot experiments and antibodies

We collected cells in lysis buffer (25 mm Tris, pH 7.4, 150 mm NaCl, 6 mm EDTA, 10% glycerol and 1% Triton X‐100) containing protease inhibitors (1 mm Pefabloc and 1.7 μg/mL aprotinin). Lysates were incubated on ice for 15 min and then centrifuged for 10 min at 10 000 ***g*** at 4 °C to clear extracts. Protein concentration was determined using the bicinchoninic acid Protein Assay Kit (Pierce). Protein extracts (30 μg) were loaded on a SDS/PAGE and transferred onto a poly(vinylidene difluoride) membrane. Western blots were performed using anti‐SCD antiserum (previously described in 10), anti‐phospho‐AKT Ser473 (CST 9271), anti‐pY99 (sc‐7020; Santa Cruz, Heidelberg, Germany), anti‐phospho‐PLCγ Tyr783 (CST 2821), anti‐PDGFRβ (sc‐432; Santa Cruz) anti‐phospho‐STAT3 Tyr705 (CST 9145), and anti‐β‐actin (A‐5441; Sigma) antibodies. We used secondary antibodies coupled with the horseradish peroxidase (HRP; Cell Signaling Technology, Leiden, the Netherlands).

### RNA extraction and RT‐qPCR

We used the RNeasy mini kit (Qiagen, Hilden, Germany) to extract total RNA following the manufacturer instructions. One microgram of RNA was subjected to reverse transcription using the M‐MLV‐RT enzyme (Thermo Fisher Scientific). Quantitative PCR analyses were performed using the following oligonucleotides: CTGTGGAGCCACCGCTCTTAC and GTTGAAGTTGATGTGCCAGCGG for human *SCD*; TCGACAATGGCAGCATCTAC and ATCCGTCTCCACAGACAAGG for the housekeeping gene *RPLP0*, as described previously 10, 26.

### Fluorescence recovery after photobleaching (FRAP)

AG01523 cells were seeded in Lab‐Tek II chambers (30 000 cells/chamber) 1 day prior to serum‐starvation for 24 h. Cells were next treated with PDGF‐BB (25 ng/mL) and/or SCD inhibitor (20 μm) for an additional 24 h. Membrane sphingomyelin was stained by incubating cells with mCherry‐lysenin (1.25 μm) for 30 min at room temperature 27. Cells were then rapidly washed and directly examined at the ZeissLSM510 confocal microscope set at 37 °C (XL/LSM incubator; Zeiss, Zaventem, Belgium), using Plan‐Apochromat 63x/1.4 oil immersion objective. Fluorescence recovery after photobleaching (FRAP) was performed as described in 27, 28.

### Statistical analysis

Student's *t* test was used to calculate statistical significance. All experiments were performed three times unless otherwise stated.

## Results

### PDGF increases SCD expression and the MUFA/SFA ratio

**Figure 1:**
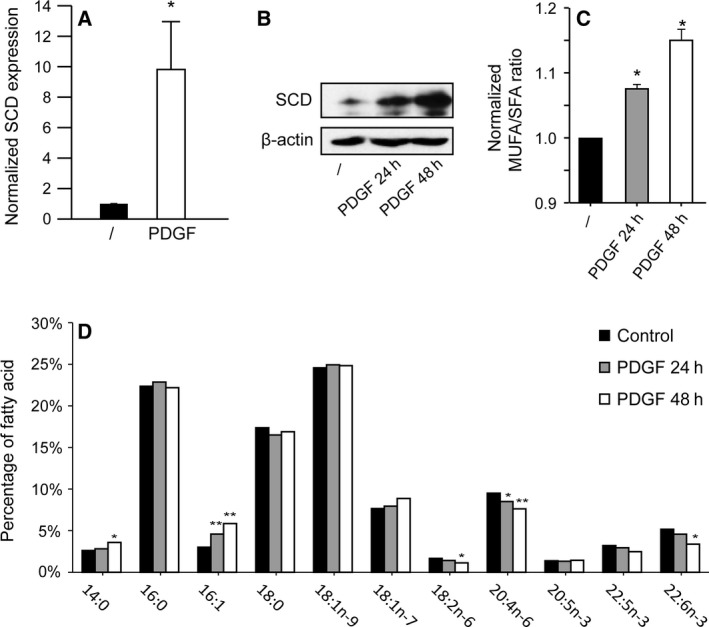
PDGF increases SCD expression and the MUFA/SFA ratio. Human fibroblasts were serum‐starved for 48 h and then treated with PDGF‐BB (25 ng/mL) for 24 h or 48 h before lysis. (A) The expression of *SCD* was measured by RT‐qPCR and divided by the expression of a housekeeping gene, *RPLP0*. (B) The SCD protein was detected by western blot using an anti‐SCD serum. (C) Lipid extraction and gas chromatography were performed as previously described 22. To calculate the MUFA/SFA ratio, we divided the sum of 16 : 1 and 18 : 1 cis fatty acids present in cells by the sum of 16 : 0 and 18 : 0. The results were normalized. The means of three (GC) or four (RT‐qPCR) independent experiments with SEM are shown. A unilateral Student's *t* test was performed for the RT‐qPCR experiment. (D) Percentages of individual fatty acids that represented at least 1% of the total fatty acid content are shown. The percentages were calculated from concentrations (mg/mL). The average of three independent experiments is shown. *P < 0.05; **P < 0.01.

In a previous study, we had shown that PDGF and other growth factors regulate lipogenic enzymes, including SCD and FASN, via a PI3K‐SREBP pathway in human fibroblasts 10. Accordingly, we had shown that PDGF stimulates the synthesis of membrane cholesterol and phospholipids. Following these experiments, we asked whether growth factors could modify the proportion of saturated and unsaturated fatty acids in cellular lipids. We first confirmed the regulation of SCD by PDGF at the mRNA and protein levels (Fig. 1A,B). Then, using gas chromatography, we analyzed the fatty acid content of fibroblasts. Cell stimulation with PDGF for 24 or 48 h in the absence of serum significantly increased the MUFA/SFA ratio (Fig. 1C). The palmitoleate/palmitate and oleate/stearate ratios increased by 32.6% ± 8.1% and 10.0% ± 4.0%, respectively, upon PDGF stimulation for 24 h (*n* = 7; *P* < 0.05). Several polyunsaturated fatty acids were decreased in PDGF‐treated cells, as a result of dilution of these essential fatty acids by newly synthesized SFA and MUFA (Fig. 1D). These results show that the up‐regulation of SCD expression by PDGF correlates with an increased proportion of MUFA in fibroblasts.

### SCD is important for the proliferation of fibroblasts in response to PDGF

**Figure 2:**
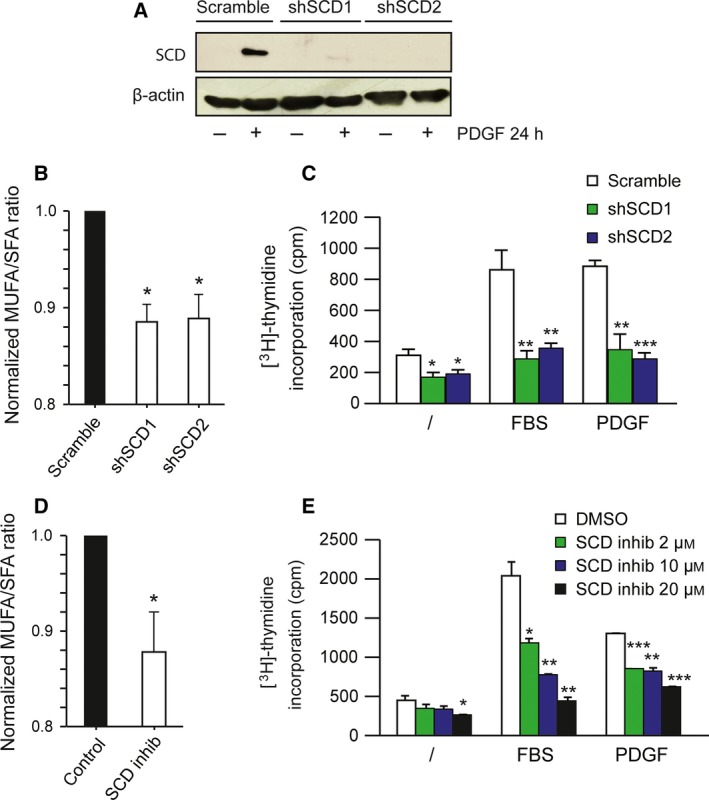
SCD knockdown or inhibition repress cell proliferation. (A–C) Human fibroblasts were infected with lentiviral particles that express shRNA targeting SCD (shSCD1 or shSCD2) or control shRNA (scramble). (A) Cells were serum‐starved for 24 h before treatment with PDGF‐BB (25 ng/mL) for 24 h. Proteins were extracted and SCD expression was detected by western blot using an anti‐SCD serum. (B) Cells were collected for fatty acid analysis as described in Fig. 1. (C) Cells were serum‐starved for 48 h and treated with FBS (10%) or PDGF‐BB (25 ng/mL) for 24 h. [^3^H]‐thymidine incorporation was measured. The result corresponds to the mean of three independent experiments with SEM. (D) Cells were treated with PDGF‐BB and SCD inhibitor (20 μm) or vehicle (DMSO) for 24 h prior to fatty acid analysis. (E) Human fibroblasts were serum‐starved for 24 h and treated with SCD inhibitor (2, 10 and 20 μm) or vehicle alone (DMSO) in the presence or absence of FBS (10%) or PDGF‐BB (25 ng/mL). [^3^H]‐thymidine incorporation was measured. The result corresponds to the mean of three independent experiments (with SEM). *P < 0.05; **P < 0.01; ***P < 0.001 compared to the corresponding control.

Next, we asked whether SCD was required for proliferation of fibroblasts in response to PDGF. To this aim, we performed experiments using two shRNAs targeting SCD (shSCD1 and shSCD2) in human fibroblasts. First, we checked the ability of the two shSCD to repress SCD expression. PDGF strongly induced SCD expression in cells expressing control shRNA. This effect of PDGF was almost completely abolished in the presence of either shSCD1 or shSCD2 (Fig. 2A). We next tested whether shSCD affected the levels of MUFA and SFA in cells. As expected, we observed that the MUFA/SFA ratio was significantly decreased when SCD expression was knocked down with shSCD1 and shSCD2 (Fig. 2B). These results demonstrate that shSCD efficiently reduced SCD expression and the Δ9 desaturase total activity in human fibroblasts.

Next, we tested whether SCD down‐regulation could influence the proliferation of normal fibroblasts in response to FBS or PDGF. While basal proliferation was slightly reduced when SCD was knocked down, the response to FBS or PDGF was blunted in the presence of shSCD (Fig. 2C). To confirm that this effect was due to the loss of SCD activity and not to an off‐target effect of shRNA, we used a pharmacological inhibitor of SCD 18, 29, which efficiently reduced the MUFA/SFA ratio (Fig. 2D). In agreement with our shRNA results, SCD inhibition reduced the proliferative response of fibroblasts to FBS and PDGF (Fig. 2E). These results demonstrate that SCD is required to stimulate the proliferation of normal fibroblasts by these growth factors.

### Loss of SCD expression or SCD inactivation has no effect on PDGFR signaling

**Figure 3:**
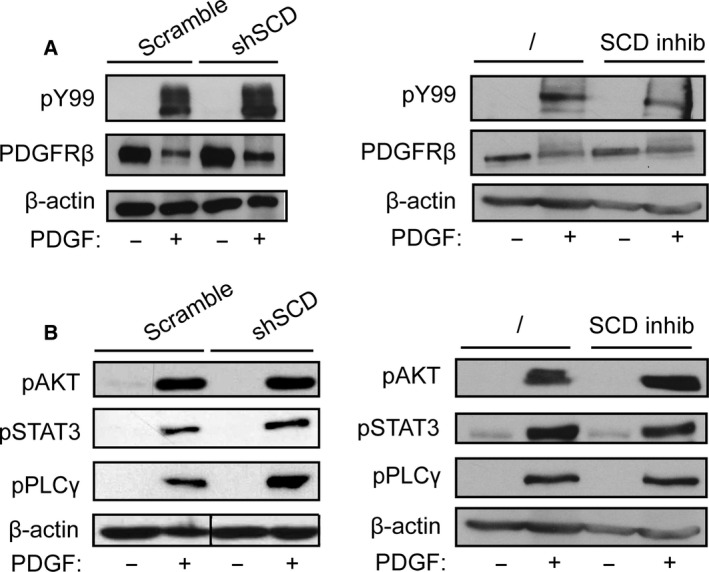
PDGFR signaling is not affected by SCD knockdown or inactivation. Human fibroblasts were infected with lentiviral particles that express either shSCD1 or control shRNA (scramble) or were treated with the SCD inhibitor (20 μm) or vehicle (DMSO) for 24 h in the absence of serum. Cells were treated with PDGF‐BB (25 ng/mL) for 15 min and proteins were extracted. Western blot experiments were performed using (A) anti‐phospho‐tyrosine (pY99), anti‐PDGFRβ, and (B) anti‐phospho‐PLCγ, anti‐phospho‐AKT, anti‐phospho‐STAT3, or anti‐β‐actin antibodies. Similar results were obtained after up to 24 h of stimulation with PDGF‐BB (data not shown).

Since SCD knockdown or inactivation blocked fibroblast response to PDGF, we hypothesized that PDGF receptor (PDGFR) expression or activity could be altered. To test this possibility, we performed western blot experiments on lysates obtained from cells that expressed shSCD and were stimulated or not with PDGF for a short period of time (15 min). We measured the expression of PDGFRβ as well as its phosphorylation, reflecting its activity. shSCD did not affect PDGFRβ expression and phosphorylation (Fig. 3A, left panel). We confirmed the absence of effect on PDGFRβ expression and phosphorylation using the SCD inhibitor (Fig. 3A, right panel). These results demonstrate that the reduction in SCD expression or activity did not alter the level of expression of PDGFR and did not interfere with its activation in response to PDGF stimulation.

Next, we assessed whether SCD inhibition could affect PDGFR signaling by measuring the phosphorylation of key PDGF‐signaling mediators, such as AKT, phospholipase Cγ, and STAT3 1. Again, western blot experiments did not show any reproducible change in PDGFR signaling in cells treated with either shSCD or SCD inhibitor (Fig. 3B). These results suggest that SCD is not required for PDGFR signal transduction.

### Palmitate and stearate accumulation mimics the loss of SCD in the proliferative response to PDGF

**Figure 4:**
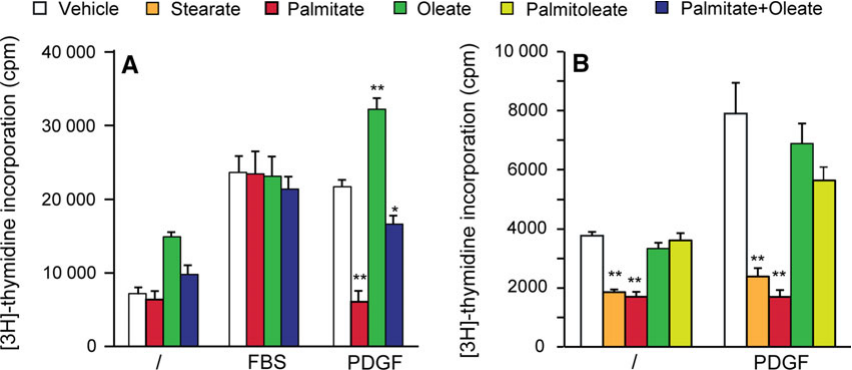
Accumulation of saturated fatty acids blocks the proliferative response to PDGF. Human fibroblasts were serum‐starved for 24 h before treatment with 0.2 mm palmitate, stearate, palmitoleate or oleate alone, or both palmitate and oleate in the presence or absence of FBS (10%) or PDGF‐BB (25 ng/mL) for 24 h. Solutions were prepared from sodium salts in A and neutralized fatty acids in B. Statistics were performed using Student *t*‐test to compare fatty acid‐treated conditions to the corresponding controls. [^3^H]‐thymidine incorporation was measured. The result corresponds to the mean of three independent experiments with SEM. *P < 0.05; **P < 0.01.

We next tested whether the addition of exogenous SFA and/or MUFA had the same effects as SCD knockdown. We performed thymidine incorporation assay with human fibroblasts treated with palmitate, oleate, or both. The treatment of fibroblasts with palmitate, a major substrate of SCD, blocked the effect of PDGF on cell proliferation (Fig. 4A). Stearate had the same effect, but not oleate or palmitoleate (Fig. 4). Interestingly, when cells were treated with palmitate in combination with oleate, thus restoring the MUFA/SFA ratio, the antiproliferative effect of palmitate was lost and cells responded to PDGF as in the control condition (Fig. 4A). Thus, the addition of oleate to palmitate reversed the negative effect caused by palmitate alone, suggesting that the MUFA/SFA ratio is important for fibroblast proliferation in response to PDGF. Palmitate had no effect on serum‐stimulated fibroblasts, possibly because serum contains traces of unsaturated fatty acids and large amounts of albumin, which may sequester palmitate 30.

### PDGF stimulation and SCD inactivation have opposite effects on plasma membrane fluidity

**Figure 5:**
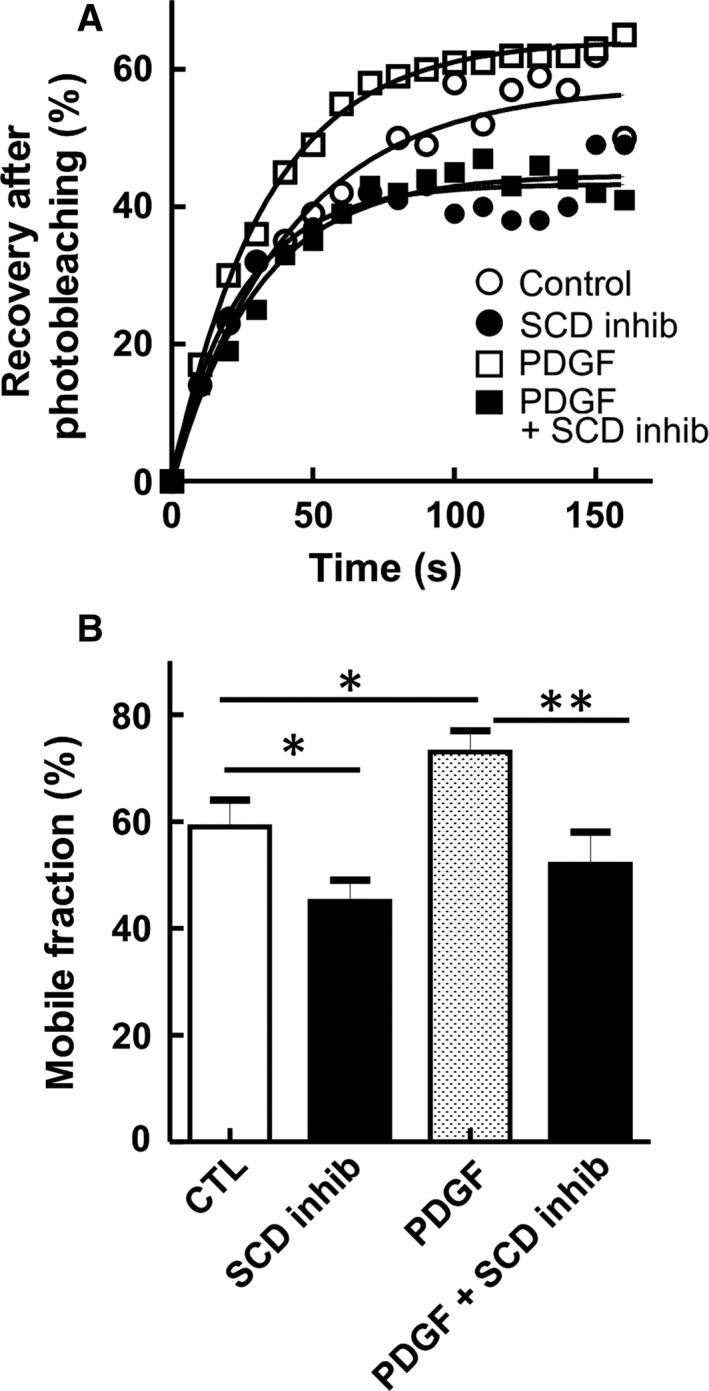
Opposite effect of SCD inhibition and PDGF stimulation on sphingomyelin lateral diffusion in the plasma membrane. Human fibroblasts were serum‐starved for 24 h and then either left untreated (open circles) or treated with PDGF‐BB alone (25 ng/mL; open squares), SCD inhibitor (20 μm; filled circles) or both (filled squares) for 24 h. Cells were then rapidly washed, labeled at 20 °C by mCherry‐lysenin, a specific probe for endogenous sphingomyelin, and directly analyzed by FRAP at 37 °C. (A) FRAP, expressed as percentage of signal before photobleaching after correction for residual fluorescence immediately after bleaching. (B) Mobile fraction values at infinite time of recovery were derived from curves presented in (A). Values are means ± SEM of 14, 22, 41, and 18 bleaching areas pooled from four independent experiments. *P < 0.05; **P < 0.01.

We then asked whether the opposite impact of PDGF stimulation and SCD inhibition on the MUFA/SFA ratio could be related to modifications of plasma membrane fluidity. To this aim, we measured the lateral diffusion of sphingomyelin by FRAP. Sphingomyelin, an abundant lipid of the outer plasma membrane leaflet, was decorated by a specific nontoxic fragment of the earthworm toxin, lysenin, fused to the red monomeric fluorescent protein mCherry 27. As shown in Fig. 5, sphingomyelin lateral diffusion in untreated fibroblasts leveled off at ~ 60%. PDGF increased the mobile fraction to ~ 75%. In contrast, upon inhibition of SCD, the mobile fraction was decreased, both in untreated and in PDGF‐treated fibroblasts. This effect is in agreement with the reduced membrane fluidity observed in human adipocytes upon SCD knockdown with siRNA 31. Altogether, our results indicate that the opposite effect of PDGF and SCD inhibitor on the MUFA/SFA ratio can be paralleled with opposite modifications of plasma membrane fluidity.

## Discussion

In the present study, we demonstrate that SCD is an important mediator of growth factor‐induced proliferation of normal human fibroblasts. First, we confirmed the strong induction of SCD by PDGF and we showed that PDGF stimulation had a significant impact on the MUFA/SFA ratio in the total cellular lipids, in agreement with an increased SCD expression. Accordingly, membrane fluidity was also increased. In addition, our results showed that SCD repression blocked the proliferative response of fibroblasts to PDGF. Together, these data indicate that growth factors do not only induce an expansion of the membrane lipid pool to promote cell proliferation but also change the cellular lipid composition and fluidity via SCD up‐regulation.

Since fibroblasts did not respond to PDGF treatment when SCD expression was reduced, we investigated whether PDGFR expression or signaling could be affected by SCD down‐regulation. Our results showed that PDGFRβ expression, phosphorylation, and signaling were unaltered by SCD down‐regulation or inactivation. These results contrasted with a previous study showing that SCD repression in prostate cancer cells led to a decrease in AKT phosphorylation due to a reduction in the phosphatidylinositol‐3,4,5‐trisposphate (PIP_3_) content in cells 32. In this study, the authors analyzed the basal activation of AKT, which was quite high, while we tested the effect of SCD down‐regulation or inhibition on PDGF‐mediated AKT phosphorylation. It is possible that in basal conditions, the level of PIP_3_ is reduced by SCD down‐regulation and becomes insufficient to activate AKT, while PDGF treatment can still induce the synthesis of a sufficient amount of PIP_3_ to induce AKT activation even when SCD expression or activity is altered.

We showed that SCD knockdown reduced the MUFA/SFA ratio, which reflected the increase in SFA and the decrease in MUFA content. In line with this result, we showed that the accumulation of palmitate and stearate mimicked the effect of SCD repression on cell proliferation in response to PDGF. Palmitate accumulation is toxic to many cell types and is known to induce ER stress 33, which we also observed in fibroblasts (data not shown). However, SCD inhibition did not induce ER stress in our cells, contrasting with published reports in other cellular models 34, 35.

We observed by FRAP that the lateral diffusion of sphingomyelin, an abundant lipid of the outer plasma membrane leaflet, was increased by PDGF. The modification in membrane fluidity correlated with a change in the unsaturated fatty acid content. Cholesterol may also affect membrane fluidity in fibroblasts, but our previous observations showed that the cholesterol/phospholipids ratio was not significantly changed by PDGF 10. Whether the change in sphingomyelin diffusion is caused by incorporation of unsaturated fatty acids directly in sphingomyelin or indirectly in phospholipids that interact with sphingomyelin in membranes remains to be established. In agreement with our observations, tumor cells usually present a higher unsaturated lipid content and an increased membrane fluidity, which are associated with increased cell proliferation 36.

The role of SCD in cancer cell proliferation and survival has been established using different cancer cell lines. SCD was also proposed as a target in renal cell carcinoma and bladder cancers 35, 37. In some of these studies, the inhibition of SCD had no effect on the proliferation of normal fibroblasts 16, 17. However, in these reports, fibroblast proliferation was only measured in basal condition, without growth factor stimulation. Our results suggest that the impact of SCD inhibitors on normal cells should be carefully re‐examined in the presence of physiological mitogens. The usefulness of SCD as a tumor target is also challenged by recent evidence suggesting a tumor suppressor role of *SCD* in chronic myeloid leukemia 38.

The role of SCD is unlikely to be restricted to cells stimulated with PDGF. We demonstrated that other growth factors, such as IGF1 and fibroblast growth factors (FGF), also stimulate SCD expression in fibroblasts 10. SCD may be part of the physiological proliferation machinery in normal and cancer cells to provide the appropriate level of MUFA to growing cells. This notion is supported by the fact that SCD expression is significantly reduced in senescent fibroblasts compared to young proliferative fibroblasts 39.

In conclusion, we showed that the induction of SCD expression is crucial for PDGF‐induced normal fibroblast proliferation.

## Author contributions

ACDB, ND and JBD designed the project. ACDB, ND, ADRS, CB, AV, YL and DT acquired, analyzed and interpreted the data. ACDB and JBD wrote the paper, which was corrected and approved by all authors.
